# Acute orchiepididymitis: Epidemiological and clinical aspects: An analysis of 152 cases

**DOI:** 10.1016/j.amsu.2022.103335

**Published:** 2022-02-09

**Authors:** A. Kbirou, M. Alafifi, M. Sayah, M. Dakir, A. Debbagh, R. Aboutaieb

**Affiliations:** a Urology Department, IBN Rochd University Hospital, Casablanca, Morocco; b Hassan University 2, Faculty of Medicine and Pharmacy, Casablanca, Morocco

**Keywords:** Orchi-epididymite, Orchite, Epididymite, Management, Emergency, AO, Acute Orchiepididymitis, ATCD, Antecedents, CBEU, Cytobacteriological examination of urine, HIV, Human immunodeficiency virus, UGI, Urogenital Infections, STD, Sexually Transmitted Disease

## Abstract

**Introduction:**

Orchiepididymitis is an inflammation of the testis and epididymis. Epididymitis, orchitis, and true orchiepididymitis are all examples of orchiepididymites. They are the most frequent cause of adult acute scrotal pain.

**Objectives:**

to investigate the epidemiological, clinical, paraclinical, therapeutic, and evolutionary characteristics of acute orchiepididymitis in the urology department of University Hospital Center.

**Materials and methods:**

This is a three-year retrospective, descriptive study of 152 patients who consulted the urology department at the university hospital center for treatment of orchiepididymitis (2017–2019).

**Results:**

In our study, 152 patients were included. The average age was 49,5 years (17–82 years). The average of consultation delay was 7 days. Prostatic pathology was found to be the main medical antecedent in 18.5% of patients, and transurethral resection of the prostate was found to be the main surgical antecedent in 8.5% of cases. Clinical examination revealed that the predominant clinical symptom was painful inflammatory bursa in 94% of cases, followed by lower urinary tract disorders in 57.5% of cases, and fever in 10% of cases.

A germ was isolated in 26 cases after a systematic cytobacteriological examination of the urine (CBEU) (17%). All patients received medical treatment, and 21% of them were received urgical treatment. In 84.5% of cases, the outcome was favorable.

**Conclusion:**

Acute orchiépididymitis is a common cause for a consultation to the emergency room. Diagnosis is based on clinical examination and ultrasound. Because of the frequency of complications and sequelae that might influence fertility in the long term, it is a diagnostic and therapeutic emergence

## Highlights

Orchiepididymitis is an inflammation of the testis and epididymis, most often of infectious origin.
Acute orchiépididymitis is a frequent reason for consultation in the emergency room.
Diagnosis is based on clinical examination and scrotal ultrasound.
The early and appropriate antibiotic treatment avoided serious complications

## 1 Introduction

Orchiepididymitis is an inflammation of the testis and epididymis, most often of infectious origin. Orchiepididymites include epididymitis, orchitis and true orchiepididymitis. They are the most common cause of acute scrotal pain in adults [1].

Over 600,000 cases are predicted to be recognised in emergency departments in the United States (United States) each year, and this disease was responsible for 1 in 144 outpatient consultations for males aged 18 to 50 [1,2]. Acute orchiepididymitis caused for 28.7% of acute scrotal aetiologies in a study of 669 patients consulting for scrotal pain in Spain [3].

Due to several microbiological etiologies and risk factors, the disease has a bimodal age occurrence [3,4]. It can be difficult to distinguish between other causes of acute bursa, such as spermatic cord torsion, especially in adolescents, necessitating surgical exploration in certain cases to confirm the diagnosis.

Despite the prevalence and severity of acute orchiepididymitis, there is a study on this fascinating topic in Morocco and the Maghreb area, which might be explained by the taboo against sexually transmitted diseases, which are one of the main causes of acute orchiepididymitis.

This study elucidated the epidemiological profile of acute orchiepididymitis by documenting its clinical, paraclinical, therapeutic, and evolution in the university hospital center's urology department. This work has been reported according to SCARE 2020 criteria [7].

## 2 Materials and methods

This is a three-year retrospective, descriptive study that included patients with acute orchiepididymitis and consulted the urology department at our hospital from January 1, 2017 to December 31, 2019.

The quantitative and qualitative variables were epidemiological, clinical, paraclinical, therapeutic and evolutionary.

The parameters studied were:

o Epidemiological: number of cases, age,
oClinics: consultation time, history, clinical signs
oParaclinical: scrotal ultrasound, cytobacteriological examination of urine, viral serologies
oTherapeutic: medical, surgical treatment
oEvolution: favorable, complications

Surgery was indicated in the event of complications, but before any surgical procedure, patients underwent an emergency biological assessment.Furthermore, patients are given broad-spectrum antibiotics prior to any surgery.

We emphasised on careful accordance with the ethical guidelines of the university hospital center's ethics committee during data collection by obtaining patient consent, maintaining the confidentiality of the data collected, and protecting the anonymity of the patients regarding the acute orchiepididymitis.

The discontinuous values were expressed as number and percentage and compared with a Chi2 test. The differences were considered significant for a p value < 0.05.

## 3 Results

**Fig. 1:**
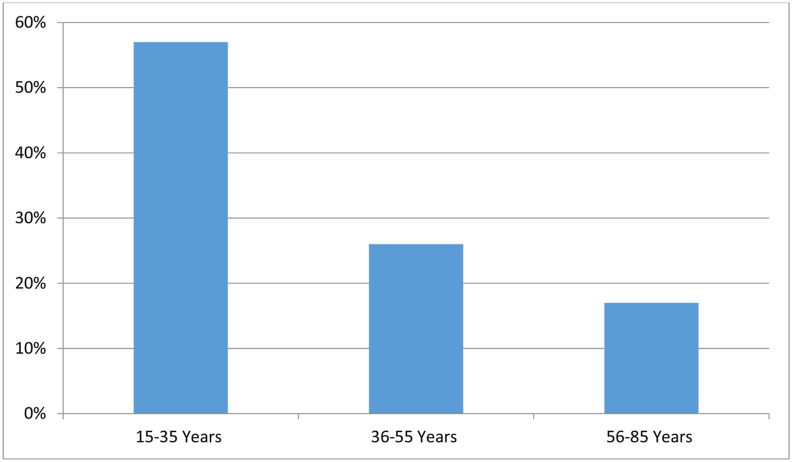
Patient distribution by age group.

152 patients were included in this study. The average age is 49.5 years with extremes between 17 and 82 years. The most affected age group is between 15 and 35 years (57%) followed by that of between 36 and 55 years (26%) and that between 56 and 85 years (17%) (Fig. 1).

The average consultation delay was 7 days with extremes between 3 and 11 days. The examination found as the main medical antecedent prostatic pathology in 28 cases (18.5%) (prostate adenoma (19 cases) and prostate cancer (9 cases)), followed by stenosis of the urethra in 11 cases (7.5%), diabetes in 7 cases (5%) and neurological bladder in 3 cases (2%). Transureteral resection of the prostate was the most common surgical antecedent in 14 cases (9.5%), followed by endoscopic urethrotomy in 8 cases (5.5%), inguinal hernia repair in 2 patients (1.5%), and cystectomy in just 1 patient (1%) (Table 1).

**Table 1:** Distribution of patients according to medical history.

ATCD		Number of cases	Percentage %	P
Medical	Prostate pathology	28	18.5	0.013
	Urethral stricture	11	7.5	0.042
	Diabetes	7	5	0.023
	Neurological bladder	3	2	0.001
Surgical	Transureteral resection of the prostate	14	9.5	0.0012
	Endoscopic urethrotomy	8	5.5	0.041
	Inguinal hernia cure	2	1.5	0.071
	Cystectomy	1	1	0.0327

Clinical examination found the large painful inflammatory bursa as the main clinical sign in 94% of cases, i.e. 143 of patients, the pain is relieved by the elevation of the testicle (preghn's sign) in 94 patients (62%) followed by lower disorders. urinary system found in 87 cases (57.5%) and fever was present in 15 cases (10%).

The cytobacteriological examination of the urine (CBEU) was systematic and isolated a germ in 26 patients (17%) (p < 0.042). Escherichia Coli was the main germ in 14 patients (9.5%), followed by *Klebsiella pneumoniae* in 6 patients (4%), *Proteus mirabilis* in 4 patients (3%) and Enterobacter fecalis in 2 patients (1.5%). However, the majority of patients, ie 126 patients (83%) (p < 0.024) had a sterile CBEU. Syphilitic viral, hepatitis B and human immunodeficiency virus (HIV) serologies were requested in 86 patients (56.5%) (p < 0.049) and revealed 4 cases of hepatitis B (3%), 2 cases of syphilis (1.5%) and 1 case of HIV infection (1%).

Scrotal ultrasound found evidence in favor of orchiepididymitis (hypertrophy and heterogeneity of the epididymis and testis, with Doppler hypervascularization) in 129 patients (85%) (p < 0.052), an associated hydrocele in 59 patients (39%) (p < 0.028), an abscessed collection in 22 cases (14.5%) (p < 0.014) and a doubt about a torsion of the spermatic cord in 9 patients (6%) (p < 0.005).

**Fig. 2:**
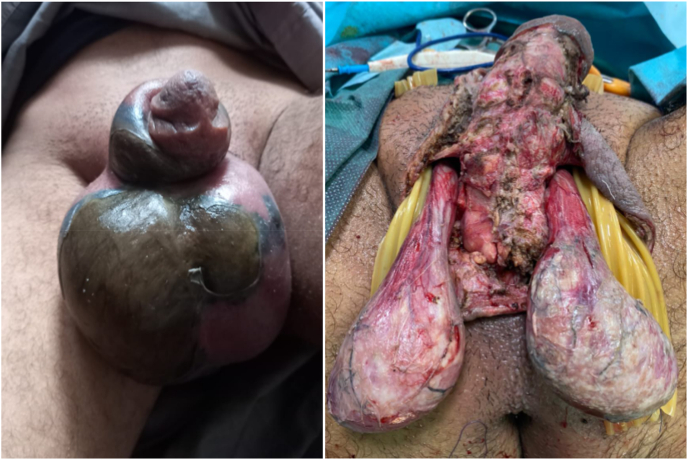
Appearance in favor of scrotal gangrene in a 45-year-old diabetic patient whose starting point was AO aggravated by the use of non-steroidal anti-inflammatory drugs. The patient was undergoing surgical scrotal necrosectomy.

All patients received medical treatment based on analgesics and antibiotics, while 32 patients (21%) received surgical treatment consisting of drainage of a scrotal abscess in 16 cases (10.5%) (p < 0.041), a suspicion of torsion of the spermatic cord in 9 patients (6%) (p < 0.037) and scrotal necrosectomy in the event of necrotizing fascist in 7 patients (5%) (p < 0.045) (Fig. 2)). The outcome was favorable in 128 patients (84.5%) (p < 0.012) while 24 of the patients (15.5%) (p < 0.045) presented complications (scrotal abscess (10.5%) and necrotizing fascitis. (5%)) (Table 2).

**Table 2:** Distribution of patients according to the evolution.

Evolution		Number of cases	Percentage (%)	P
Favorable		128	84.5	0.012
Complications	Scrotal abscess	16	10.5	0.041
	Necrotizing fascitis	7	5	0.045

## 4 Discussion

Genitourinary emergencies in men are estimated to be between 0.5% and 2.5% of all emergency room visits, while there is little data that specifically reports the incidence of acute scrotum as a complaint [1]. The etiologies of acute scrotum are multiples, they include traumatic, infectious, inflammatory and idiopathic etiologies which can have repercussions on fertility and male sexuality.

This study deals with a frequent pathology that is seldom recognised and investigated not just in our context but also in the literature, and it demonstrates the crucial importance of early diagnosis of sexually transmitted infections and sex education in preventing acute orchiepididymitis and its complications. Although acute orchiepididymitis can be innocuous, our study illustrates the potential complications that can occur in the case of a delayed diagnosis and the absence of appropriate therapy.

This study was the first in our country to deal specifically with acute orchiepididymitis. A recent European study estimated the incidence of epididymitis at 2.45 per 1000 [[2], [3], [4], [5], [6], [7], [8]]. Epididymitis refers to inflammation of the epididymis. It mainly affects young adults, with a peak in frequency between 20 and 40 years [9,10]. It does not know a preferential side but it is bilateral in nearly 10% of cases [9,10]. Most often, it is secondary to an infection whose usual route of dissemination is retrograde deferential. We can thus distinguish two periods of puberty at age 35, when epididymitis is frequently sexually transmitted [2,3]. *Chlamydia trachomatis* and Neisseria gonorrhoeae are the main germs involved [2,3]. Before puberty and after 35 years, epididymitis is part of the usual urogenital infections (UGI), for which enterobacteria are often responsible [2,3]. A urological pathology, in particular an obstacle on the lower urinary tract, is then readily at the origin of the infection [5]. Orchitis, which is rarer, refers to inflammation of the testicle. The route of dissemination is either hematogenous, particularly viral (the most typical example is mumps orchitis), or direct contact with an epididymitis (it is then a true orchi-epididymitis) [9,10]. patients was 49.5 years old, which was explained by the existence of urological pathologies mainly associated with prostatic pathology (23%) but the most affected age group was between 15 and 35 years. Hoosen reported on a series of 144 cases, an average age of 24 years and 93% of these patients were under 35 years [15]. 5 years which was explained by the existence of urological pathologies associated mainly with prostatic pathology (23%) but the age group most affected was between 15 and 35 years. Hoosen reported on a series of 144 cases, an average age of 24 years and 93% of these patients were under 35 years [14]. 5 years which was explained by the existence of urological pathologies associated mainly with prostatic pathology (23%) but the age group most affected was between 15 and 35 years. Hoosen reported on a series of 144 cases, an average age of 24 years and 93% of these patients were under 35 years [15].

The picture of an acute epididymitis linked to a retrograde ductal infection (sexually transmitted or part of a “classic” UGI) was described clinically [2]. Most often, the onset of an epididymitis is acute and hard. a day or two, it is sometimes abrupt or more progressive [9]. In our series the average consultation time was 7 days. The patient often reports severe pain in the bursa, typically radiating along the spermatic cord to the inguinal region and relieved by lifting of the testicle (Prehn's sign) [3]. The interview looks for voiding disturbances which are inconstant and indicate urethritis or associated acute prostatitis [8,9]. The attending physician should seek the notion of urethral discharge in favor of urethritis, which more readily evokes the gonococcus than *C. trachomatis*, allowing him to administer emergency treatment [12]. The fever, often high, is inconstant [[8], [9], [10], [11], [12]]. Clinical examination looks for an enlarged bursa and thickened, inflammatory scrotal skin [2]. Palpation is difficult due to the severity of the pain, which is sometimes impossible or uninterpretable in the presence of a hydrocele [14,15]. It shows an enlarged and painful epididymis, the initial signs of which are located in the tail [15,16]. The spermatic cord is often tense, enlarged and painful. The testicle, initially normal, is then affected by the inflammatory process [16,17]. Palpation then perceives a painful mass without being able to distinguish the epididymis and the testis (true orchiepididymitis). The digital rectal examination looks for prostate pain suggestive of associated acute prostatitis [8,9].

Acute inflammatory scrotal pain was the primary manifestation in our work. Berger et al. had reported in a series of 69 cases as the main clinical signs acute scrotal pain (68%), followed by urinary disorders (32%) and fever (8.6%) [16]. At the paraclinical level, the cytobacteriological examination of the urine (CBEU) (second test) and the search for sexually transmitted germs are systematic except when a “classic” UGI is manifest (elderly patient, known or manifest urological pathology, surgical intervention or manipulation. recent lower urinary tract) [[10], [11], [12]]. Currently, urethral sampling is carried out by simple swabbing in search of Neisseria gonorrhoeae, because the endourethral sample with scraping is replaced by the search for Chlamidaie trachomatis on the first urine jet by gene amplification technique (PCR) and the sperm culture does not add anything to the usual samples [[8], [9], [10], [11], [12], [13], [14], [15], [16], [17]]. An infectious agent is demonstrated in approximately 70% of epididymitis [9,10].

Sexually transmitted germs are responsible for 35% of epididymitis, most of which occurs from puberty to 35 years [3]. The most frequent are *Chlamydia trachomatis*, the discovery of which has upset the approach to epididymitis, and Neisseria gonorrhoeae [13]. In some rare situations, rare germs can be isolated in 10% of cases, depending on the patient's immune status [18,19]. These are Brucella, *Mycobacterium tuberculosis*, Haemophilus influenzae, salmonella or viruses (herpes varicella zoster [HVZ], cytomegalovirus [CMV], Ebstein-Barr virus [EBV], adenovirus, coxsackiesvirus, echovirus, mumps virus, virus rubella) [20,21]. Exceptionally, parasitic or mycotic attacks are described, especially in severely immunocompromised patients.

The scrotal Doppler ultrasound is systematically performed in the event of clinical suspicion of orchi-epididymitis allowing both to confirm the diagnosis and to eliminate the differential diagnoses (torsion, necrotic tumor) and complications (abscess, vascular anomaly) and allows also to orient the diagnosis towards the nature of the germ in certain particular situations [18,19]. It is indeed very useful for monitoring and must be repeated during the course [18,19].

Regarding treatment, non-specific measures combine bed rest, analgesics and the jockstrap. The choice of antibiotic therapy depends on the clinical and bacteriological orientation. In the acute phase, the inflammation increases the penetration of antibiotics into the infected epididymis [21]. Treatment with tetracyclines or fluoroquinolones for a period of 3–4 weeks is recommended in case of suspected STD with simultaneous management of the or partners [22,23]. However, in the presence of signs in favor of urological disease, the combination trimethoprim-sulfamethoxazole or fluoroquinolones is more readily used for an identical period [22,23]. Of course, a urological assessment of the lower urinary tract is essential (ultrasound, IVU with voiding images and possibly retrograde cystography) [[24], [25], [26]]. Hospitalization in an intensive care unit with administration of a double intravenous antibiotic therapy combining a third-generation cephalosporin with an aminoglycoside, are reserved for severe situations secondary to diagnostic delay and in the event of insufficient or ill-suited treatment [27,28]. Surgical treatment of orchiepididymitis is indicated in the face of clinical signs of seriousness (funiculitiss, loss of anatomical landmarks, scrotal skin fixation), signs on scintigraphy or echo-doppler suggesting testicular ischemia [29,30].In the long term, there may be repercussions on male fertility, which was explained either by epididymal obstruction by fibrous nuclei in bilateral involvement or by impairment of spermatogenesis in the testicular parenchyma [31,32].

## 5 Conclusion

Orchiepididymitis are frequent reasons for emergency consultations. All ages are concerned and the aetiologies are multiple, dominated by urogenital infections in young subjects and the existence of progressive urological pathology in elderly patients, which explains the frequency and diversity of the germs isolated. The diagnosis is clinical, the ultrasound allows confirmation of the diagnosis and the highlighting of complications. Early and appropriate treatment avoids these complications which can lead to sequelae, thus affecting male fertility.

## Funding

We have any financial sources for our research.

## Provenance and peer review

Not commissioned, externally peer-reviewed.

## Declaration of competing interest

All authors disclose any conflicts of interest.
